# Supporting Autistic Children's Participation in Research Studies: A Mixed-Methods Study of Familiarizing Autistic Children with A Humanoid Robot

**DOI:** 10.1177/23969415251332486

**Published:** 2025-04-24

**Authors:** Carly McGregor, Elisabeth von dem Hagen, Christopher Wallbridge, Jenna Dobbs, Caitlyn Svenson-Tree, Catherine RG Jones

**Affiliations:** 1 Wales Autism Research Centre, School of Psychology, Cardiff University, Cardiff, UK; 2 Centre for Artificial Intelligence, Robotics and Human-Machine Systems (IROHMS), Cardiff University, Cardiff, UK; 3 School of Computer Science and Informatics, Cardiff University, Cardiff, UK

**Keywords:** Autism, robots, humanoid, familiarization, participation

## Abstract

It is important that autism research is inclusive and supports the participation of a wide range of autistic people. However, there has been limited research on how to make studies accessible for autistic participants. This mixed-methods study explored how to promote the comfort of autistic children in research, using the specific example of visiting a research lab and meeting a humanoid robot. In Phase 1, 14 parents of autistic children were interviewed about how their child could be made comfortable during a lab visit, including different approaches for familiarizing their child with the robot. In Phase 2, autistic children of the parents in Phase 1 (*n* = 10) visited the lab and completed familiarization activities with a humanoid robot. The opinions of the children and their parents about the children's experiences were recorded. Using reflexive thematic analysis, five overarching themes reflected how to best support autistic child participants. These themes encompassed elements of particular relevance to robot studies but also many practices of general relevance to participating in research: (1) preparation is key, (2) consideration of environmental factors, (3) using familiarization, (4) a supportive and engaged researcher, and (5) individualized approaches. Based on our findings, we report preliminary and generalizable best-practice recommendations to support autistic children in a research setting and promote positive experiences.

The importance of promoting inclusivity and positive experiences for autistic individuals who participate in research is becoming increasingly recognized (e.g., Gowen et al., 2019; Haas et al., 2016; Pellicano et al., 2017). Many autistic characteristics, such as sensory sensitivities (Yuan et al., 2022), the need for routine (Louis-Delsoin et al., 2024), and communication difficulties (Askari et al., 2015), may present as barriers to study participation. Co-occurring features, such as anxiety (e.g., van Steensel et al., 2011) and intolerance for uncertainty (e.g., Boulter et al., 2014), can also limit inclusion. Not only do researchers have a responsibility to ensure participation is as comfortable and as positive as possible, but inclusive research practices are key to ensuring that autism research is not limited to a narrow range of autistic people.

Guidance exists for researchers on how to promote the comfort of autistic participants during research participation. These recommendations include environmental considerations, such as considering the sensory environment and providing breaks during testing, as well as the provision of clear instructions, both on what will happen during the testing session and how to find the venue (Gowen et al., 2019). Guidelines framed specifically for autistic adults also highlight the importance of accessible consent processes and offering multiple modes of participation (Nicolaidis et al., 2019). Related to this is the recommended use of research passports, which can help autistic people communicate their needs and preferences to researchers (Ashworth et al., 2021). Researchers have also developed recommendations for working with autistic children who are non-speaking or who have intellectual disabilities, and stress the importance of monitoring participants' energy levels and paying attention to behavioral signs that the participant may not wish to continue testing (McKinney et al., 2021). However, existing insights and recommendations are based on surveys of the literature, reflections on researchers' own experiences, or consultation with autistic adults or parents of autistic children about their previous experiences with research. To date, no research study has used preparation for or participation in a research study to directly generate insights from autistic people or family members.

Research involving children necessarily involves additional considerations compared to adult participants (Fargas-Malet et al., 2010), and this is particularly the case for children with additional needs. For autistic children, a relevant consideration is how they might experience the novel equipment that is often a feature of experimental studies. Autistic children are often invited to take part in studies that include specialist equipment, including neuroimaging (Rafiee et al., 2022), eye tracking (Papagiannopoulou et al., 2014), virtual reality (Chen et al., 2024), and robots (Alabdulkareem et al., 2022). Research with robots, particularly with a focus on human–robot interaction (HRI), has become increasingly popular in recent years. Robots are often favored as a data collection tool because of the experimental control they afford (e.g., Chevalier et al., 2020; Giannopulu et al., 2014; Golliot et al., 2015). Human experimenters may send unintentional messages via subconscious modifications to their voice or facial expressions, but this can be tightly controlled in HRI studies (Huijnen et al., 2019). Other studies focus on HRI specifically, using robots in interventions to improve the well-being of autistic children (e.g., Boccanfuso et al., 2017; Fachantidis et al., 2020; Huijnen et al., 2021; Kajopoulos et al., 2015).

Autistic children can find meeting a novel robot in an experimental setting difficult, leading to discomfort, distress, and even participant withdrawal (Huijnen et al., 2021; Short et al., 2017). There are a range of factors that may make the experimental setting uncomfortable for autistic children. Many of these, including the disruption to routine and the introduction to unfamiliar people and settings, are generalizable to many types of study. However, there are likely additional challenges that occur when meeting an unfamiliar robot. Indeed, researchers have identified the engineering challenge of designing robot interactions in a way that is both engaging and perceived as nonthreatening for children (Scassellati et al., 2012). Specific anxiety about the robot has been highlighted as a driver of discomfort in previous HRI studies with autistic children (e.g., Di Nuovo et al., 2020; Petric et al., 2017). Difficulties with tolerating the uncertainty of the robot's behaviors (e.g., Boulter et al., 2014) and sensory discomfort (e.g., Kirby et al., 2022) are additional factors that may be relevant.

Familiarization phases are a potential way of helping autistic children overcome discomfort when meeting a robot. A familiarization phase is an initial phase of an experiment designed to introduce participants to key aspects of the methodology, and is used in HRI research (Wallbridge et al., 2023). Previously, including a familiarization phase in an HRI study reduced the withdrawal rate of autistic child participants from 87.5% to 10.5% (Petric et al., 2017), indicating they can have a significant impact. Our systematic review of the familiarization strategies used by researchers to introduce autistic participants found that a wide variety of approaches were used (Wallbridge et al., 2023), such as showing the participant what the robot can do, or having the participant and the robot participate in an activity together. However, the majority of studies that reported using a familiarization phase provided limited detail. As such, the understanding of familiarization approaches remains limited. Other researchers who use specialized equipment that would likely be unfamiliar to children, such as magnetic resonance imaging (MRI) scanners, have successfully taken steps to generate bespoke guidance for familiarizing autistic children with their study equipment (Tziraki et al., 2021). Although familiarization methods do exist within the field of HRI (Wallbridge et al., 2023), there has been no specific exploration of the success of these techniques, including the perspectives of parents and autistic children.

In the current study, our primary aim was to investigate how to promote the comfort and enjoyment of autistic children in research, using the specific example of meeting a humanoid robot in a laboratory setting. By using a real-world example, we wanted to generate nuanced and ecologically valid insights that could complement previous consultation work (e.g., Gowen et al., 2019; Nicolaidis et al., 2019). Additionally, we wanted to focus on the voices of autistic children and their parents. As such, we used a two-phase, mixed-methods design that involved a preparatory interview and a study visit. In the first phase, we conducted online semi-structured interviews with parents to discuss how to promote their children's comfort in a research setting, alongside a more specific exploration of the suitability of various robot familiarization techniques. In the second phase, their children visited our lab to meet a humanoid robot, giving them an opportunity to explore the effectiveness of different familiarization methods. Both the parents and children provided feedback on the child's experience of meeting the robot.

## Methods

### Participants

Fourteen parents and their autistic children were recruited through an advertisement on social media. The children were aged 6–11 years old, had a clinical diagnosis of autism, and had no significant physical disability that would limit their ability to interact with a humanoid robot.

All parents participated in Phase 1, an online interview, and were invited to take part in Phase 2, which occurred in the lab an average of 11.2 days (*SD* = 7.7) after Phase 1. Ten of the 14 parents took part with their autistic child in Phase 2. Three other children chose not to participate: one decided not to take part on the morning of the study, and two chose not to participate upon arrival at the lab, but before beginning the study.

Parents completed the lifetime version of the Social Communication Questionnaire (SCQ) (Rutter et al., 2003). Scores ranged from 18 to 36 (*M* = 25.8, *SD* = 6.1), with a score of 15 or higher indicating that a child might be autistic. While all parents reported in the SCQ that their children were “able to talk using short phrases or sentences,” three parents answered that they were unable to have a “to a to and fro ‘conversation’ with [their child] that involves taking turns or building on what [they] have said.” Participant demographics for each phase of the study are shown in Table 1.

**Table 1.** Participant Demographics.

Sample Characteristics	*n*	Range	*M*	*SD*
*Phase 1—Parents (n = 14)*				
Gender				
Female	13			
Male	0			
Unknown	1			
Race/ethnicity				
White British	11			
Mexican	1			
White and Black African	1			
Unknown	1			
Age (years)		31–44	37.3	3.2
*Phase 2—Parents (n = 10)*				
Gender				
Female	9			
Male	0			
Unknown	1			
Race/ethnicity				
White British	7			
Mexican	1			
White and Black African	1			
Unknown	1			
Age (years)		36–44	36.7	3.0
*Phase 2—Children (n = 10)*				
Gender				
Female	2			
Male	7			
Unknown	1			
Race/ethnicity				
White British	7			
Mexican	1			
Indian, White, and Black African	1			
Unknown	1			
Age (years)		6.4–11.3	8.8	1.5

Parents received a £15 shopping voucher for participating. Children received a certificate and a sticker. The study was approved by the [redacted for anonymity]. Each parent provided written informed consent and the children provided verbal assent.

### Materials

#### Humanoid Robot

**Figure 1.**
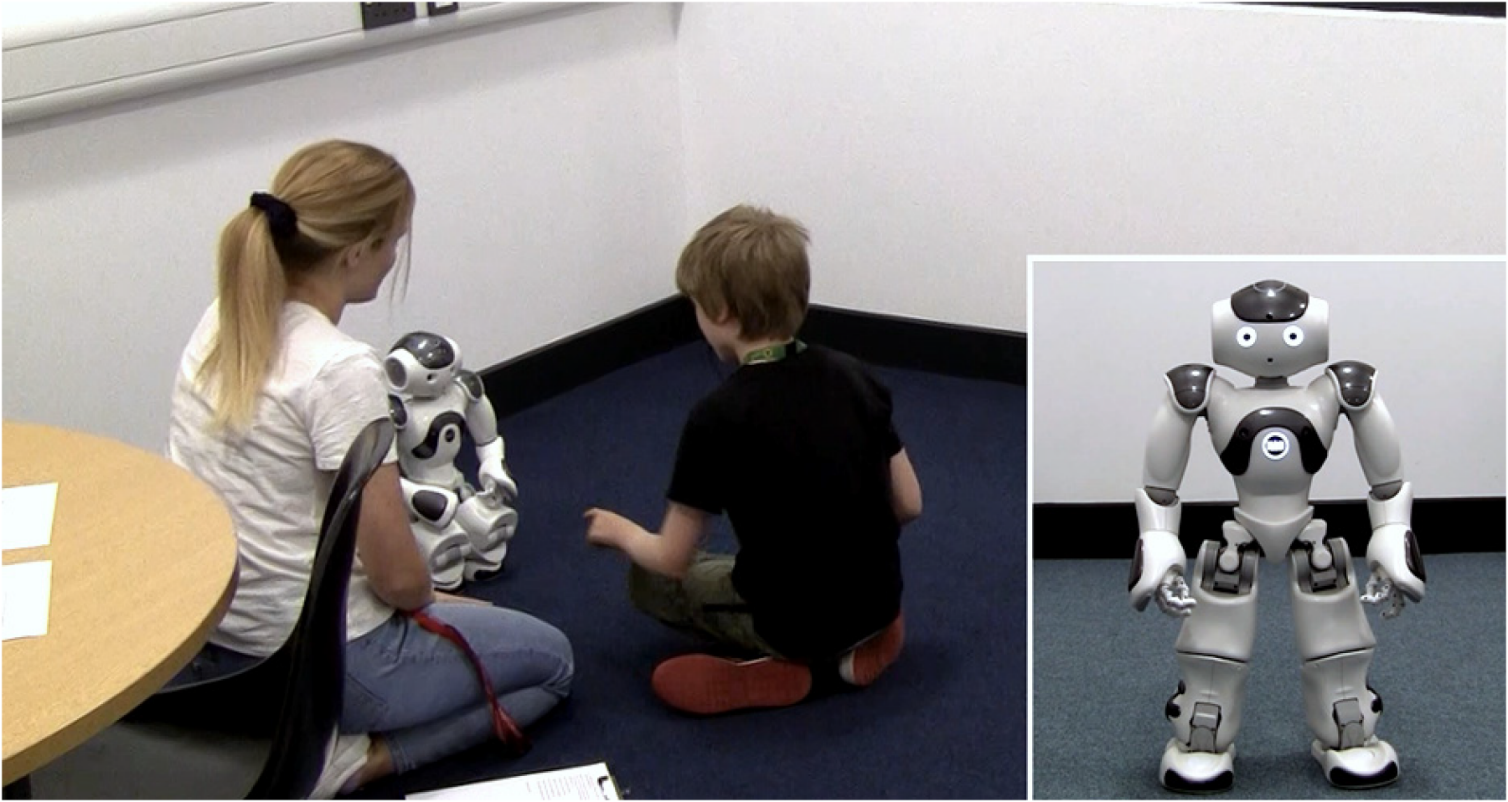
Humanoid Robot National Audit Office (NAO) by SoftBank Robotics.

*Note:* A member of the research team and a child participant interacting with the NAO robot. A clearer photo of the NAO robot is shown on the right.

We used an NAO robot from the United Robotics Group, which is commonly used in studies with autistic participants (Wallbridge et al., 2023). NAO robots are 57.4 cm tall, with tactile sensors and speakers, and are capable of speech, movement, and playing music (Figure 1). They connect to a computer via Wi-Fi and are controlled using Choregraphe (Pot et al., 2009) software. Further details of the NAO robot can be found elsewhere (e.g., Puglisi et al., 2022). We named the NAO robot Russell and used male pronouns.

#### Familiarization Approaches

The familiarization approaches (see Table 2) were discussed with parents in the Phase 1 interviews and used with the children in the Phase 2 lab visits. The approaches were based on a subset of those identified by Wallbridge et al. (2023) that were considered suitable for the NAO robot and/or the current method. These approaches were developed in Choregraphe Version 2.8.7.4 (Pot et al., 2009), and are available as part of a Git repository.^1^ Additional details are located in the Supplemental Material.

**Table 2.** Familiarization Approaches were Discussed with Parents in the Phase 1 Interviews and Used with the Children in the Phase 2 lab visit.

Familiarization Approach	Description
Capability demonstration	
Wake-up sequence	The robot gradually wakes up and introduces itself.
Song and dance	The robot plays a song and does a dance along with it.
Stimulus and response	
Question and answer session	The robot asks the child several questions, and also provides its own answers.
Following game	The robot asks the child to do several simple movements.
Mindful breathing	The robot leads the child through a mindful breathing exercise.
Static exploration	
Free exploration	The robot is “powered off” and the researcher invites the child to touch the robot.
Guided exploration	The robot is “powered off” and the researcher asks the child to touch specific parts of the robot.

**Capability demonstration.** This approach gradually displayed each of the robot's capabilities, while the child watched passively. This was achieved by the robot conducting a wake-up sequence (Part 1), which was followed by a song and dance routine (Part 2).

**Stimulus and response.** This approach enabled two-way interactions between the robot and the child. Three different stimulus and response exchange options were available: a question and answer session, a following game where the child completed simple instructions given by the robot, and a mindful breathing exercise.

**Static exploration.** This approach enabled the child to explore the robot using touch without the robot responding. Two types of static exploration were available: free exploration, in which the researcher invited the child to touch the robot, and guided exploration, in which the researcher guided the child through touching different parts of the robot in the context of giving the robot a “check-up.”

#### Phase 1—Parent Interview

A semi-structured online interview was conducted to explore parents’ perspectives on how their child could be supported to visit our lab to meet a humanoid robot. Parents were shown a video of the NAO robot and its capabilities were described. The first part of the interview included a discussion of how the parent's child might feel about meeting the robot, alongside topics about how to promote the child's comfort during the visit. For the second part of the interview, parents were shown videos of the NAO robot enacting the capability demonstration (wake-up sequence; song and dance) and stimulus and response. Static exploration was described to parents verbally. Each familiarization approach was followed by a discussion about how the parent thought their child would feel about the approach. For approaches that had multiple implementation options (e.g., free exploration or guided exploration), parents were asked to reflect on which option might best suit their child. The interview ended with a focus on parents’ opinions about sharing information about their child's needs with researchers before a study, and how they thought this should be carried out.

#### Phase 2—Parent Ratings and Interview

Parents used Likert scales to indicate how comfortable they thought their child was during each familiarization approach, and how much their child enjoyed each approach. At the end of the session, parents also reported how much their children enjoyed themselves overall. All scales ranged from 1 to 5, with 1 representing the *most negative* response and 5 the *most positive*.

A semi-structured interview was also conducted with the parent in which they elaborated on their Likert scores. Questions were also asked about the appropriateness of the duration of each approach and the number of approaches used.

#### Phase 2—Child Interviews

Child participants were asked about their experience of spending time with the robot using three questions, answered using a 5-point Likert scale where 1 was the *most negative* response and 5 was the *most positive*. The questions were, “Do you think Russell is friendly?,” “Do you think Russell is happy?,” and “How happy would you be to play with Russell again?.” The first two questions were adapted from the Robotic Social Attributes Scale (Carpinella et al., 2017). Participants were first given three options and told to select the one they most agreed with (e.g., friendly, in the middle, and unfriendly). If participants selected friendly, then they were asked if they thought the robot was very friendly or just a little friendly. Analogous options were provided for unfriendly, whereas in the middle was recorded as is. Answers could be expressed verbally, through pointing, or head shaking/nodding.

Children who could answer open-ended questions also completed a short semi-structured interview where they were asked about what they liked or disliked about the robot, what their favorite activity was (song and dance portion of the capability demonstration, stimulus and response approach, or static exploration), and what other games that the robot should learn to play. If open-ended questions were not accessible to a child, they were instead shown pictures representing the different activities and asked to point at their favorite activity, and any activities they did not like.

## Procedure

**Figure 2.**
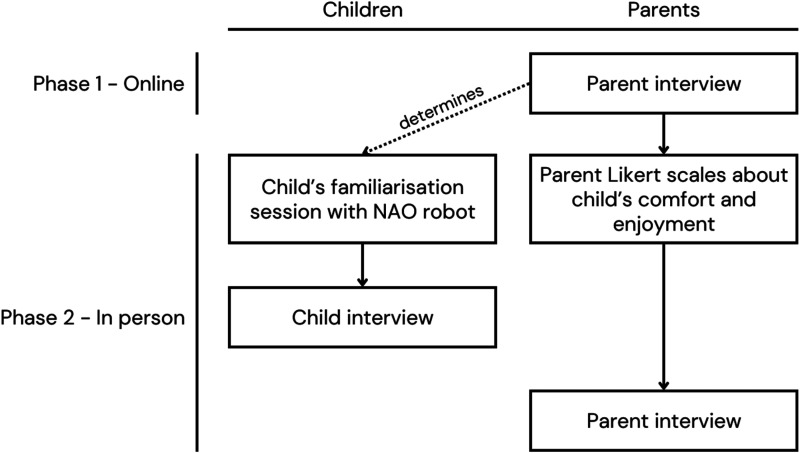
Order of Activities within the Study Phases.

The order of the different parts of the study is represented visually in Figure 2.

### Phase 1—Parent Interview

Interviews were conducted online in Microsoft Teams by one of two researchers and lasted an average of 38 min (range: 28–52 min). The interviews were recorded and transcribed. Researchers showed parents the familiarization methods by sharing their screen and playing a prerecorded video.

### Phase 2—Child and Parent Lab Visit

As suggested by a parent in Phase 1, prior to the lab visit, each family was sent a storyboard that explained in simple terms what the child would be doing, accompanied by photographs. Parents could also request additional preparation materials during Phase 1. Two parents requested a video of the researchers introducing themselves, and another requested a letter from the researchers assuring the child that they were allowed to bring their comfort objects.

During the lab visit, one researcher worked directly with the child while a second researcher operated the robot. Before entering the testing room, the child was shown a photograph of the robot and was given the opportunity to ask questions. Upon entering the room, the child could choose where they sat.

The child was then presented with the three familiarization approaches in the order: capability demonstration, stimulus and response, and static exploration. This fixed order was chosen as the level of interaction with the robot increases across the approaches. The specific implementation of each approach was predetermined in the Phase 1 parent interview. After all familiarization approaches were completed, the researcher asked the child about their experience with the robot using Likert scales and the child interview.

During the child's session with the robot, their parent sat in an observation room with the second researcher, where they observed the testing session through a one-way mirror. Parents completed the Likert interviews regarding their child's perceived enjoyment and comfort after each familiarization approach. This was followed by the semi-structured interview, which lasted an average of 11 min (range: 7–15 min).

## Data Analysis

Parents’ semi-structured interviews in Phases 1 and 2 were recorded and automatically transcribed in Microsoft Teams, then later checked for accuracy and anonymized. We used the method of thematic analysis to explore our data, drawing in particular on the approach of reflexive thematic analysis (RTA; Braun & Clarke, 2021b). With this method, we identified patterns and generated themes across both sets of interviews, with information from transcripts of Phases 1 and 2 treated with equal weight. The pragmatic nature of our research aim, with its focus on what supports autistic children in a research setting, informed our approach to the RTA. Our analysis was embedded in an experiential and realist framework, where we aimed to capture participant’s perspectives and insights as directly expressed within the data. Aligned with this, our approach was inductive, meaning that our data coding and subsequent theme creation was driven by the data, rather than being shaped by pre-existing constructs about good research practices for autistic children. We focused on a semantic level of meaning in the data, which meant we explored what was being said directly rather than searching for latent, implicit meaning. An RTA approach also meant that our subjectivity and experiences with supporting autistic individuals were conceptualized as a useful tool to scaffold our understanding of the data, rather than as a threat to the validity of the results (Braun & Clarke, 2021a).

For the TA, two researchers independently coded all parent interview transcripts in NVivo 12 (Dhakal, 2022), coding in reverse order of one another. All interviews were double coded. Periodic checks were made to ensure consensus between coders, agreeing upon what data was relevant and how the codes should be labeled. Codes were then merged across the two coders to create a single dataset, with each coder independently merging half of the coded interviews. Where the codes used were different between coders, the coder selected the one that they considered most accurately represented the parents' comments. Both researchers refined the codes together, identifying and merging similar codes across the dataset. The coders then established initial thematic groupings together, which were further refined through discussion with the wider research team. Likert scales and child interviews were not integrated into the themes, and were analyzed separately.

Both coders were nonautistic and were not parents. However, both coders were raised with autistic family members, and one had several years of experience working with autistic children, which meant they had professional experience in strategies for supporting autistic children in daily life. One of the coders was not British and they were sensitive to the possibility of cultural misreading. However, the collaborative coding strategy provided reassurance. It was also reflected that one coder conducted most interviews while the other did not collect any data; this created a balance between having a richer but more subjective position and having a position of greater objectivity.

The use of two coders allowed the exploration of different subjective interpretations of the data, driven by distinct perspectives and backgrounds, enabling the development of a richer conceptualization of participants’ responses. Our pre-existing intention to develop guidelines for researchers affected the framing of our themes as we wanted them to have salience as potential action points. In generating information meant to be used by other researchers, the two primary coders and the wider research team subjectively and intentionally use their viewpoints as researchers to thematically interpret the dataset.

The children's answers to the open-ended questions in the interview were transcribed and summarized; data were too limited for the TA. The Likert scales for both parents and children were explored using summary statistics.

## Community Involvement Statement

Autistic community members were not involved in the development of this research study.

## Results

### Thematic Analysis

**Figure 3.**
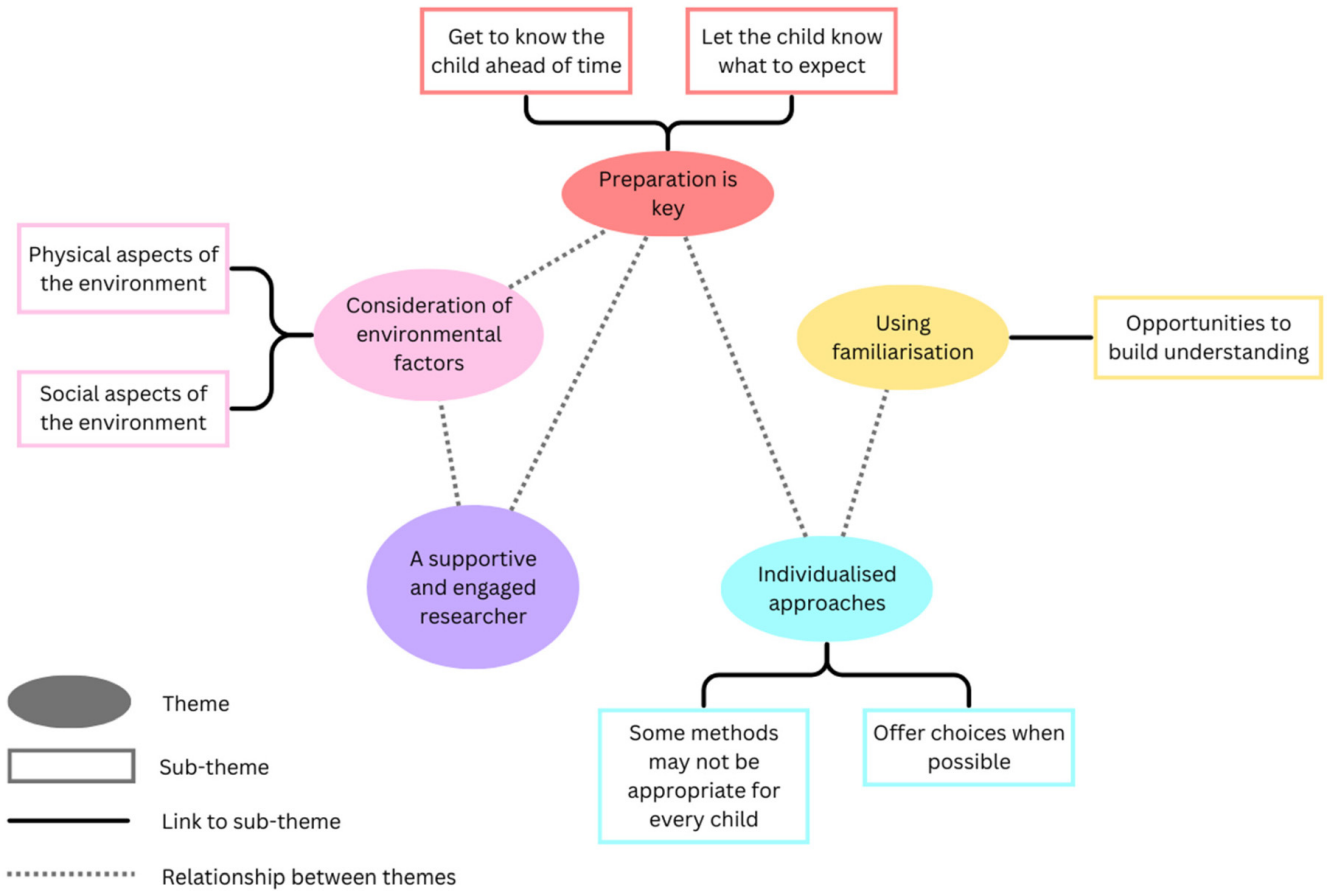
Thematic Map of Parents' Perspectives on How to Effectively Engage Autistic Children in Research with Humanoid Robots.

Five main themes were generated from the parents’ interviews: (1) preparation is key, (2) consideration of environmental factors, (3) using familiarization, (4) a supportive and engaged researcher, and (5) individualized approaches. Four of the themes had distinct subthemes. The relationships between the themes are depicted in Figure 3.

#### Preparation is Key

Parents reflected that sharing information between the family and researchers before the child's visit was an important part of ensuring their child's comfort, because it helped the research team prepare for how to best support their child and gave the child a clearer idea of what to expect. Two subthemes were generated within this theme: *Get to know the child ahead of time*, and *Let the child know what to expect*.

**Get to know the child ahead of time.** Many parents explained that their children had differences in their communication style or additional conditions that impacted their interactions (e.g., anxiety, attention deficit hyperactivity disorder, and speech delay). Some offered guidance on how to communicate with their child to help facilitate positive interactions:

[My child] communicates through a third person, so to speak, or he prefers to be told in the third person. So inside him, he has somebody called “Fluffy Puppy.*” Now, if you want him to do something, if you ask Puppy to do it, he finds it far easier. (Parent #14)

*This term has been anonymised

Some parents reported that sensory sensitivities, anxieties, or fears could cause significant distress for their children. A parent noted the importance of researchers knowing about these before meeting their child:

I think it's better to do this “cause you can kind of understand what's gonna frighten [my child].” (Parent #8)

**Let the child know what to expect.** Many parents expressed that their child feels anxious when dealing with the unknown. One parent explained a routine their child regularly completed before going somewhere new:

He will look at videos of the place and what there is to do there, who is there, and what things look like … Otherwise … he's got to do that all before he actually gets through to what he needs to do. (Parent #14)

- Parent #14

The storyboard each family received was cited by many as a valuable tool in helping prepare their child. As one parent explained:

He was a bit, “What do I do? Wait, what is my purpose to be here?” When I read [the storyboard] it was like, “Oh, right, I’m here to have fun.” (Parent #10)

Some parents explained that it was important for them to have a complete understanding of what would happen, as it would enable them to help prepare their child.

#### Consideration of Environmental Factors

This theme was represented by two subthemes; parents thought that both physical and social environmental factors had the potential to make their child uncomfortable during their visit.

**Physical aspects of the environment.** Anxiety related to being in an unfamiliar space was a common concern among parents. Some indicated that this would be a greater source of anxiety for their child than the robot:

He might be less comfortable with the setting than the robot. The robot, he won't have a problem with. It might just be the setting it's used in. (Parent #10)

Some parents also reported that visual aspects of the space, such as the room appearing too clinical, could cause their child to be nervous.

**Social aspects of the environment.** Interpersonal aspects of the study were another concern, particularly in relation to the child's anxiety around strangers. Many parents highlighted that their children found it difficult to effectively communicate with people they did not know.

He does struggle to speak to most people, unless he knows them really well. He kind of shuts down and withdraws, and just kind of shakes his head. So he just gets really anxious and nervous and doesn't know how to express himself properly. (Parent #16)

This relates closely to the theme *Preparation is key*, as some parents suggested this anxiety might be reduced if their child could see photographs of the researchers before their visit.

Some parents said that their children may also feel self-conscious, awkward, or embarrassed if they sensed they were being watched. This was both in reference to the child being aware of their parent and a researcher observing them through a one-way mirror, and the sentiment that their child may feel embarrassed engaging in certain activities in the presence of any audience. One parent suggested that this feeling of awkwardness can be reduced by limiting the number of people who are observing the child.

Some parents commented that their child may feel more comfortable if their parent was in the testing room, providing a “familiar face.” However, other parents said that their child might find this distracting:

… I think he's more likely to focus if I'm not there. So I've taken him to an optician's appointment, and because I was there, he got more distracted. He was trying to interact with me and not the optician. (Parent #13)

In summary, parents felt that there were several elements of the study environment unrelated to the robot that could impact how their child feels and behaves.

#### Using Familiarization

Parents viewed familiarization as an important factor in promoting a successful study session, with many saying that their child would initially need time to “warm up” to the robot. Some parents also highlighted the importance of the child forming a “connection” with the robot.

Some parents were less concerned about their child being anxious around the robot, and instead thought their child might lose interest in it. Potential causes included the child's short attention span, the robot being less responsive than expected, or robots not being one of their child's interests. The duration of the familiarization activities was therefore deemed an important consideration.

Ultimately, parents felt that taking part in a research study should be fun for their children. Some stressed this as being crucial, indicating that their child was resistant to participating in things they didn't enjoy.

Because [if] you mentioned school or homework … he just completely shuts down. But if he thinks it's gonna be fun, yeah, he'd be all for it and probably get really involved. (Parent #16)

Within this theme, there was one subtheme related to the benefits of increasing the child's understanding of the robot.

**Opportunities to build understanding.** Parents indicated that effective familiarization methods were ones that built the child's understanding of the robot. Some parents highlighted activities that gave the child an opportunity to explore the robot on a mechanical level. Other parents stressed the importance of activities that showed the robot's capabilities or nature, saying their child may initially worry that the robot was unfriendly or dangerous:

He might be a little, like, daunted at first … because of the things we see about robots being like, good robots and bad robots … He’ll probably get more comfortable when he realizes that it's not any sort of threat. (Parent #13)

In summary, parents thought it was important for the familiarization session to build the child's trust and understanding in the robot, while still keeping them engaged.

#### A Supportive and Engaged Researcher

Parents noted having a researcher lead their child through the interactions helped the child understand that it was safe for them to follow the robot's instructions. As one parent explained:

Notice that sort of about halfway through, [the researcher] didn't have to repeat what Russell said. [My child] was just going off what Russell said. So that's sort of like, a sort of gradual level of trust … “I can do it and nothing bad's gonna happen … It's safe to do.” (Parent #9)

Similarly, some parents reported that their child may have been anxious about what they were allowed to do, such as how to touch the robot without damaging it, causing the child to be more cautious and reserved. Parents suggested that this could be overcome by having the researcher give explicit permission, clear boundaries, and demonstrate what to do.

This theme closely relates to the theme *Preparation is key*, as having a good understanding of the child's anxieties, communication style, and needs enables researchers to better support them during the study. It also relates to the *Consideration of environmental factors*, as the research team has the potential to be a source of support for the child's anxiety.

#### Individualized Approaches

Parents described that the most effective familiarization method may depend on the child. There were two subthemes: *Some methods may not be appropriate for every child*, and *Offer choices when possible*.

**Some methods may not be appropriate for every child.** Many parents said some familiarization options may not be appropriate for their child due to their age or perception of their own maturity. One parent, when reflecting on the song choices, explained why they chose the robot dance over the nursery rhyme options:

I think that he would think that the other two are quite babyish, and he thinks that he's a lot more grown-up than that. (Parent #4)

Others reported some methods would be too complex for their child, which could cause them to lose interest in the robot. Similarly, many children experienced difficulties with speech or movement, which that would make it difficult for them to complete certain tasks.

Some parents suggested their child would not need to experience all of the familiarization approaches, as they anticipated their child would feel comfortable around the robot almost immediately. Conversely, others said that their child would need them all, and may even need additional time with a particular activity, or more time with the robot in general, to feel comfortable.

This subtheme is also closely related to the theme *Preparation is key*, as having this information ahead of time enables researchers to make adjustments to the familiarization protocol where necessary.

**Offer choices when possible.** Parents suggested that, when possible, it would be beneficial to let their children choose how they would like to interact with the robot. While this was not the case for every child, many parents said that their child would feel comfortable communicating their needs and preferences with the researchers*.* Parents also reflected on the benefits of giving children choices in less formal ways. For example, children were allowed to sit anywhere in the room during the study, whilst the robot remained stationary. Parents said this enabled their child to choose how far away from the robot they would like to be:

It's like staying in the one spot … He can move away if he needs to… (Parent #15)

In summary, individualized approaches were considered beneficial and reflected the children's abilities and preferences. This is closely related to the theme of *Using familiarization*, as the most appropriate way to acclimate each child to the robot varied.

### Parent and Child Preferences

#### Phase 1—Parent Selection of Familiarization Activities

The 14 parents who participated in the Phase 1 interview selected which stimulus and response and static exploration activities would be most appropriate for their child. The following game was the most common stimulus and response choice, and guided exploration was the most common static exploration choice (see Table 3).

**Table 3.** Parent's Familiarization Choices During the Phase 1 Previsit Interview.

Familiarization Approach	Number of Parents
Stimulus and response	
Following game	9 (64%)
Question and answer	4 (29%)
Mindful breathing	1 (7%)
Static exploration	
Guided exploration	9 (64%)
Free exploration	5 (36%)

#### Phase 2—Parent and Child Preferences

Nine children completed all of the familiarization approaches, whereas one child chose to end the familiarization session after the wake-up sequence. Their parent explained the child was eager to attend another activity. Children who completed all of the familiarization activities were asked which activity was their favorite: the song and dance portion of the capability demonstration segment, the stimulus and response activity, or the static exploration. The stimulus and response activity was the most popular choice amongst the children (Table 4). One child did not have a favorite activity.

**Table 4.** Parents’ and Children's Preferred Familiarization Activities in Phase 2.

Familiarization Approach	Number of Participants
Children	
Stimulus and response	4 (50%)
Capability demonstration	3 (38%)
Static exploration	1 (13%)
Parents	
Stimulus and response	7 (78%)
Capability demonstration	1 (13%)
Static exploration	1 (13%)

The children's parents were asked which one approach would be the most helpful in familiarizing their child with the robot. Similar to the children, the stimulus and response approach was the most popular choice amongst parents (Table 4).

### Comfort and Enjoyment Ratings

**Figure 4.**
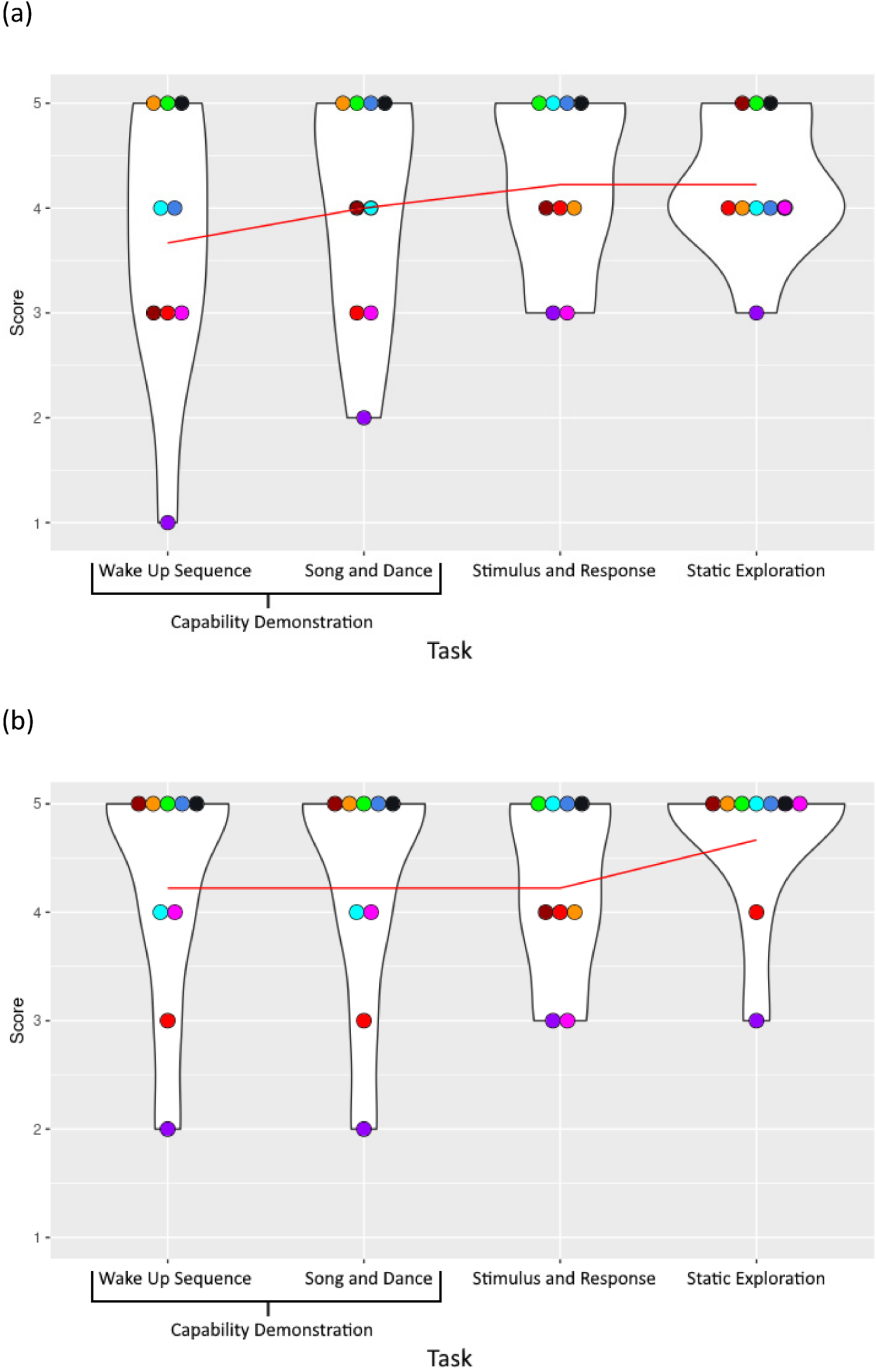
Parent Ratings of (a) Child Comfort and (b) Enjoyment During Each Familiarization Activity in Phase 2.

*Note:* Each color represents an individual child. The line shows the mean scores. For scoring, 1 = *very uncomfortable/did not enjoy* and 5 = *very comfortable/really enjoyed*.

Overall, parents rated their children as showing high levels of comfort with, and enjoyment of, the activities (Figure 4). Although statistical analysis was not appropriate with the small sample, both comfort and enjoyment improved for the group as a whole across the activities.

#### Children's Opinions about the Robot

**Figure 5.**
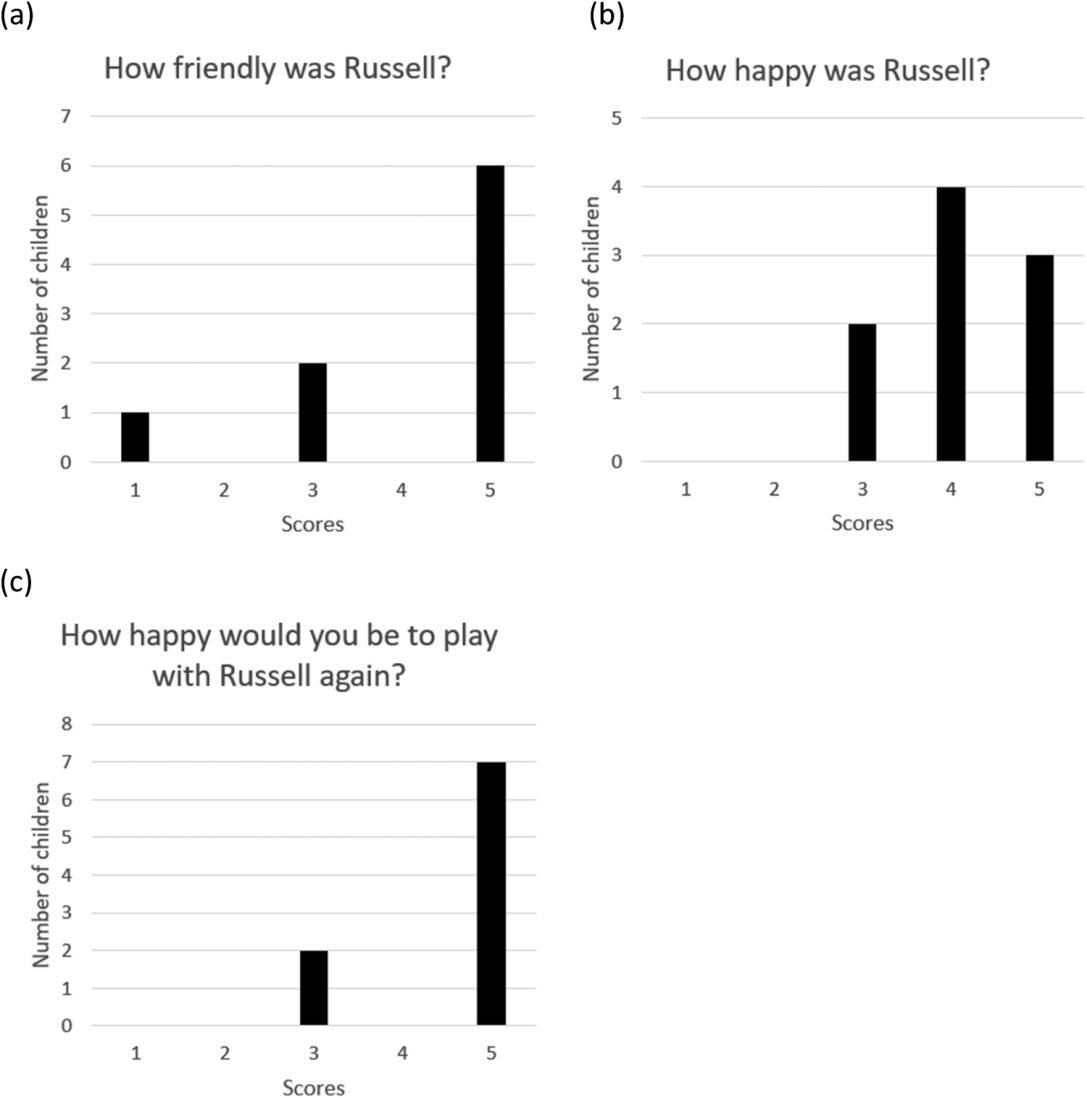
Children's Opinions About the Robot in Phase 2.

*Note:* For scoring, 1 = *most negative* and 5 = *most positive response options*.

Following familiarization, children were asked to rate how friendly the robot was (mean score of 4.1 [*SD* = 1.45]), how happy the robot was (*M* = 4.1 ± 0.78), and how happy they would be to play with the robot again (*M* = 4.6 ± 0.88) (Figure 5). Some children found these questions difficult to answer. When asked if the robot was friendly, one child said, “I don't know what that means.” Another, when asked if he thought the robot was happy, said “I can't really tell if he's happy.” The researcher offered clarification and all children made selections.

Seven children were able to verbally answer open-ended questions and were asked about what they liked/disliked about the robot. Children volunteered limited information, but most responses were positive. Some children identified their favorite thing about the robot, with responses encompassing specific features (his fingers, his waking up sequence, and his dancing), his mood (“very happy” and “fun”), and generic endorsement (“everything”). Most children did not identify anything they disliked about the robot, but one commented about the robot being a bit loud when he talked. An additional child observed that the robot's gaze followed them when they moved, which was “a little bit creepy.”

## Discussion

Using mixed-methods, we explored the most effective approaches for supporting the comfort and enjoyment of autistic children when meeting a humanoid robot in a research setting. Qualitative analysis of interviews with autistic children's parents identified five themes regarding how to best support autistic children in a research setting, using the specific example of meeting a humanoid robot: (1) preparation is key, (2) consideration of environmental factors, (3) using familiarization, (4) a supportive and engaged researcher, and (5) individualized approaches. Additional qualitative and quantitative data indicated that parents and children felt positively about the child's experience of meeting the robot and our familiarization techniques were successful. Synthesizing across our findings, we present a set of preliminary recommendations for researchers wanting to support the inclusion of autistic children in research.

In our investigation of research inclusion for autistic children, we explored three different approaches for familiarizing autistic children with a humanoid robot. Across qualitative and quantitative measurements, all familiarization methods were well received and can be recommended for use with autistic children, depending on the child's needs and the study requirements. Parents and children were generally well-aligned in their preferences for different familiarization approaches. Children preferred the stimulus and response and capability demonstration approaches over the static exploration, and parents showed a strong preference for stimulus and response activities. The stimulus and response approach was unique in that it elicited two-way interactions between the child and the robot, which may be an important factor. Static exploration, which could be very open-ended and relatively un-scaffolded, was the least preferred and may reflect the preference for routine in autistic people (e.g., Leekam et al., 2011). However, the fixed order of the approaches, chosen to enable a graded introduction, may have influenced opinions.

Despite humanoid robots potentially being a source of discomfort for autistic children (Wallbridge et al., 2023), they include properties that may be particularly appealing. Indeed, many autistic children find meeting a robot positive (e.g., Garnier et al., 2023). Both autistic adults and educators of autistic children have highlighted that the consistent and predictable nature of robots, compared to the complexity of human interaction, may make them attractive to autistic children (Alcorn et al., 2019; Silvera-Tawil & Roberts-Yates, 2018). Educators also recognized that robots could be inherently engaging to autistic children (Alcorn et al., 2019). Further, although robots may contain sensory features that some autistic children find difficult, these can invariably be adjusted to suit the child's needs (e.g., Kumazaki et al., 2022). Thus, arguably, the role of the familiarization phase is to convey these appealing properties to an autistic child, so that initial uncertainty and wariness can be replaced by confidence in the parameters of the robot.

Despite humanoid robots potentially being a source of discomfort for autistic children (Wallbridge et al., 2023), they include properties that may be particularly appealing. Indeed, many autistic children find meeting a robot positive (e.g., Garnier et al., 2023). Both autistic adults and educators of autistic children have highlighted that the consistent and predictable nature of robots, compared to the complexity of human interaction, may make them attractive to autistic children (Alcorn et al., 2019; Silvera-Tawil & Roberts-Yates, 2018). Educators also recognized that robots could be inherently engaging to autistic children (Alcorn et al., 2019). Further, although robots may contain sensory features that some autistic children find difficult, these can invariably be adjusted to suit the child's needs (e.g., Kumazaki et al., 2022). Thus, arguably, the role of the familiarization phase is to convey these appealing properties to an autistic child, so that initial uncertainty and wariness can be replaced by confidence in the parameters of the robot.

One consideration when recommending our familiarization techniques is how generalizable they would be to studies using other robots. However, the familiarization approaches used in the current study were drawn from a previous systematic review of studies that collectively used 28 different models of robots with autistic participants (Wallbridge et al., 2023). Further, the majority of studies did not use the NAO robot, as used in the current study. As such, the techniques are likely to be applicable to other robots being used in research. However, the specific execution of our familiarization techniques will depend on the capabilities of the robot and some adaptations may be required (e.g., if the robot cannot play music). Similarly, the specific features of the robot (e.g., quality of sound and fluidity of movement) may enhance or inhibit the impact of certain familiarization approaches.

While parents typically found the familiarization approaches to be effective, they also stressed the importance of beginning the familiarization process before the lab visit. Parents' reports that their children benefit from having comprehensive information ahead of time is consistent with the perspectives of autistic adults, who also value prestudy information (Gowen et al., 2019). In the current study, a parent specifically suggested we use a storyboard to explain the laboratory visit to their child, and this was used with all children. Building from this, we recommend a digestible introduction to the study is given to all child participants. This could take the form of Social Stories^TM^ (Gray & Garand, 1993), an existing storyboard framework that is widely used (Qi et al., 2018). However, our data also indicated that parents should be encouraged to suggest specific preparatory materials that may support their child (e.g., a letter that allays a concern). Supplementing introductory materials with photos and videos, framed in the context of “What to expect,” were considered useful by autistic adult participants (Gowen et al., 2019). This highlights that many needs will not be age-specific, although approaches must be age-appropriate. The benefit of preparatory materials likely reflects the elevated intolerance of uncertainty (Boulter et al., 2014; Wigham et al., 2015) and anxiety (MacNeil et al., 2009) often seen in autistic people.

Although a humanoid robot was the focus of the current study, many other studies also use novel equipment that may be difficult for autistic children. For example, sources of discomfort for autistic children in the MRI scanning environment were similar to those identified in the current study, including sensitivity to loud noises and being in an unfamiliar space (Tziraki et al., 2021). Tziraki et al. identified techniques that could help introduce autistic children to an MRI scanner that have resonance with our own findings, including learning about each child's communication style and providing families with preparatory materials. However, some techniques reflected the specific challenges of the equipment, such as showing children a miniature “toy” MR scanner and listening to the scanner noise in advance of the scanning session. These findings highlight that there will be shared sources of potential discomfort across most experimental studies, alongside study-specific considerations. Indeed, although the focus of our qualitative interview with parents was about familiarizing autistic children with a humanoid robot, discussion of the study visit elicited many comments that reflected general study features. These included the potential challenge of meeting unfamiliar people and being in an unfamiliar place. Researchers should therefore consider both the general and study-specific elements of their research when preparing autistic children and families for a research study.

Expanding on the wider relevance of the current study, many of the approaches we identified are applicable outside of research contexts. Autistic children may encounter robots or other novel equipment in clinical or educational settings (Huijnen et al., 2017; Saleh et al., 2021), with negative reactions to meeting a humanoid robot documented in both classroom and clinical settings (e.g., Di Nuovo et al., 2020; Garnier et al., 2023). Introductions to robots may need to be different in applied contexts, for example, managing expectations about access to the robot in the classroom (Silvera-Tawil & Roberts-Yates, 2018). However, many of the fundamental principles described in the current study will be applicable to these settings.

Many of the parents we interviewed indicated that their child might struggle with aspects of the study because of characteristics that were not directly related to being autistic. Several children experienced co-occurring difficulties, including attentional difficulties, specific fears, and anxiety. The high prevalence and wide range of additional diagnoses and/or traits in autistic populations (Bougeard et al., 2021; Rodriguez-Seijas et al., 2020; Rosen et al., 2018) further exemplify the importance of learning about each child before they participate in a study. Utilizing existing frameworks such as research passports (Ashworth et al., 2021) can support this aim of enabling autistic participants to share their needs and preferences with researchers. While autistic children may need familiarization more than other groups, the diversity of children's needs make it likely that *all* children would respond positively to tailored approaches to promote their comfort and enjoyment while participating in research.

### Recommendations

Utilizing themes that were generated from parent interviews, we have developed a set of recommendations for promoting the comfort of autistic children in research settings (Table 5). As illustrated in Table 5, each recommendation draws from one of the themes we generated through RTA. Reflecting our data, the recommendations are focused on steps that can be taken before the testing phase of the study. Importantly, the recommendations are not specific to studies that include robots, but are broadly applicable to studies with autistic children. Many of our suggestions complement recommendations offered by Gowen et al. (2019) on how to make studies more accessible for autistic participants, particularly how to prepare participants and selecting comfortable, sensory-friendly testing spaces.

**Table 5.** Recommendations for Supporting Autistic Children in Research Settings Based on Themes Generated from the Current Study.

		Description	Associated Theme
1	Testing space	Choose a testing space that is child-friendly and that avoids common sensory triggers, such as bright lights or loud background noise.	Consideration of environmental factors
2	Learn about the participant	Provide an opportunity for parents to share information about their child before the study session.	Preparation is key
3	Adjust protocol	Adjust the familiarization and study protocol based on the information received from the parents.	Individualized approaches
4	Preparation materials	Send families preparation materials to help the children better understand what to expect when they come for the study.	Preparation is key
5	Supportive researcher	Have a member of the research team present who is prepared to support the child throughout the study.	A supportive and engaged researcher

### Limitations and Future Directions

In the current study, the preparation materials sent to families prior to the lab visit prepared most children for their familiarization sessions, but three children did not want to visit the lab for the study. This suggests that more work is needed to identify the most effective preparation materials.

Additional work can also be done to collect richer feedback from autistic children. We used simple questions to be inclusive of children with limited verbal communication. However, this approach limited the depth of feedback gained from children with more advanced communication abilities. In future, a tiered approach, where the data collected from children is adapted to suit their abilities, may enable children to be better represented. There are also creative qualitative methods that can be used to interview autistic children, including more visual and embodied child-led interviews (Lewis et al., 2024). It is likely that these approaches would have enabled better inclusivity of children with alternative communication styles or more limited receptive language.

Expanding on the appropriateness of the child questions, two children found it difficult to decide how happy or friendly the robot was. These questions were adapted from an existing questionnaire about how people perceive robots (Carpinella et al., 2017). However, autistic children often understand or define friendships differently than non-autistic children (Petrina et al., 2014), which may have explained the challenges some children experienced. It is also unknown whether the perceived happiness or friendliness of a robot are key factors in influencing how much autistic children like robots. Additional research is needed to determine what attributes of robots influence autistic children's opinions. This will enable researchers to more accurately evaluate robots’ interactions in accordance with what is important to autistic children.

The current study was not an exhaustive exploration of familiarization methods that have been used in research with autistic children and humanoid robots (see Wallbridge et al., 2023), and only one type of robot was used. Further research is needed to evaluate the effectiveness of other approaches across a wider range of robots.

## Conclusion

Using mixed-methods, we worked with parents and autistic children to determine how to promote children's comfort and well-being during research, using the specific example of a laboratory visit to meet a humanoid robot. We found that our robot familiarization methods were well received, and also gained valuable insights into generalizable techniques for supporting positive participant experiences. We have summarized our findings into a set of general recommendations that we hope will support future researchers in delivering inclusive research with autistic children.

## Supplemental Material

sj-docx-1-dli-10.1177_23969415251332486 - Supplemental material for Supporting Autistic Children's Participation in Research Studies: A Mixed-Methods Study of Familiarizing Autistic Children with A Humanoid Robot Supplemental material, sj-docx-1-dli-10.1177_23969415251332486 for Supporting Autistic Children's Participation in Research Studies: A Mixed-Methods Study of Familiarizing Autistic Children with A Humanoid Robot by Carly McGregor, Elisabeth von dem Hagen, Christopher Wallbridge, Jenna Dobbs, Caitlyn Svenson-Tree and Catherine RG Jones in Autism & Developmental Language Impairments

## Notes

1. https://github.com/CWallbridge/Familiarisation.

## References

[bibr1-23969415251332486] Alabdulkareem A. Alhakbani N. Al-Nafjan A. (2022). A systematic review of research on robot-assisted therapy for children with autism. Sensors, 22(3), 944. 10.3390/s22030944 35161697 PMC8840582

[bibr2-23969415251332486] Alcorn A. M. Ainger E. Charisi V. Mantinioti S. Petrović S. Schadenberg B. R. Tavassoli T. Pellicano E. (2019). Educators’ views on using humanoid robots with autistic learners in special education settings in England. Frontiers in Robotics and AI, 6, 107. 10.3389/frobt.2019.00107 33501122 PMC7805648

[bibr3-23969415251332486] Ashworth M. Crane L. Steward R. Bovis M. Pellicano E. (2021). Toward empathetic autism research: Developing an autism-specific research passport. Autism in Adulthood: Challenges and Management, 3(3), 280–288. 10.1089/aut.2020.0026 36605365 PMC8992899

[bibr4-23969415251332486] Askari S. Anaby D. Bergthorson M. Majnemer A. Elsabbagh M. Zwaigenbaum L. (2015). Participation of children and youth with autism spectrum disorder: A scoping review. Review Journal of Autism and Developmental Disorders, 2(1), 103–114. 10.1007/s40489-014-0040-7

[bibr5-23969415251332486] Boccanfuso L. Scarborough S. Abramson R. K. Hall A. V. Wright H. H. O’Kane J. M. (2017). A low-cost socially assistive robot and robot-assisted intervention for children with autism spectrum disorder: Field trials and lessons learned. Autonomous Robots, 41(3), 637–655. 10.1007/s10514-016-9554-4

[bibr6-23969415251332486] Bougeard C. Picarel-Blanchot F. Schmid R. Campbell R. Buitelaar J. (2021). Prevalence of autism spectrum disorder and co-morbidities in children and adolescents: A systematic literature review. Frontiers in Psychiatry, 12, 744709. 10.3389/fpsyt.2021.744709 PMC8579007 34777048

[bibr7-23969415251332486] Boulter C. Freeston M. South M. Rodgers J. (2014). Intolerance of uncertainty as a framework for understanding anxiety in children and adolescents with autism spectrum disorders. Journal of Autism and Developmental Disorders, 44(6), 1391–1402. 10.1007/s10803-013-2001-x 24272526

[bibr8-23969415251332486] Braun V. Clarke V. (2021a). Can I use TA? Should I use TA? Should I not use TA? Comparing reflexive thematic analysis and other pattern-based qualitative analytic approaches. Counselling and Psychotherapy Research, 21(1), 37–47. 10.1002/capr.12360

[bibr9-23969415251332486] Braun V. Clarke V. (2021b). Thematic Analysis: A Practical Guide. Sage.

[bibr10-23969415251332486] Carpinella C. Wyman A. Perez M. Stroessner S. (2017). The Robotic Social Attributes Scale (RoSAS): Development and Validation. 254–262. 10.1145/2909824.3020208

[bibr11-23969415251332486] Chen J. Hu J. Zhang K. Zeng X. Ma Y. Lu W. Zhang K. Wang G. (2024). Virtual reality enhances the social skills of children with autism spectrum disorder: A review. Interactive Learning Environments, 32(5), 2321–2342. 10.1080/10494820.2022.2146139

[bibr12-23969415251332486] Chevalier P. Kompatsiari K. Ciardo F. Wykowska A. (2020). Examining joint attention with the use of humanoid robots – A new approach to study fundamental mechanisms of social cognition. Psychonomic Bulletin & Review, 27(2), 217–236. 10.3758/s13423-019-01689-4 31848909 PMC7093354

[bibr13-23969415251332486] Dhakal K. (2022). NVivo. Journal of the Medical Library Association: JMLA, 110(2), 270–272. 10.5195/jmla.2022.1271 35440911 PMC9014916

[bibr14-23969415251332486] Di Nuovo A. Bamforth J. Conti D. Sage K. Ibbotson R. Clegg J. Westaway A. Arnold K. (2020). An Explorative Study on Robotics for Supporting Children with Autism Spectrum Disorder during Clinical Procedures. *Companion of the 2020 ACM/IEEE International Conference on Human-Robot Interaction*, 189–191. 10.1145/3371382.3378277

[bibr15-23969415251332486] Fachantidis N. Syriopoulou-Delli C. K. Zygopoulou M. (2020). The effectiveness of socially assistive robotics in children with autism spectrum disorder. International Journal of Developmental Disabilities, 66(2), 113–121. 10.1080/20473869.2018.1495391 PMC8132921 34141373

[bibr16-23969415251332486] Fargas-Malet M. McSherry D. Larkin E. Robinson C. (2010). Research with children: Methodological issues and innovative techniques. Journal of Early Childhood Research, 8(2), 175–192. 10.1177/1476718X09345412

[bibr17-23969415251332486] Garnier P. Martel K. Dachez J. Audry P. Bourgoin P. Stawinski F. (2023). Educators’ perspectives on working with a humanoid robot in a French preschool class for autistic children. Journal of Research in Special Educational Needs, 23(3), 163–174. 10.1111/1471-3802.12588

[bibr18-23969415251332486] Giannopulu I. Montreynaud V. Watanabe T. (2014). PEKOPPA: A Minimalistic Toy Robot to Analyse a Listener-Speaker Situation in Neurotypical and Autistic Children Aged 6 Years. *Proceedings of the Second International Conference on Human-Agent Interaction*, 9–16. 10.1145/2658861.2658872

[bibr19-23969415251332486] Golliot J. Raby-Nahas C. Vezina M. Merat Y.-M. Beaudoin A.-J. Couture M. Salter T. Côté B. Duclos C. Lavoie M. Michaud F. (2015). A Tool to Diagnose Autism in Children Aged Between Two to Five Old: An Exploratory Study with the Robot QueBall. *Proceedings of the Tenth Annual ACM/IEEE International Conference on Human-Robot Interaction Extended Abstracts*, 61–62. 10.1145/2701973.2701975

[bibr20-23969415251332486] Gowen E. Taylor R. Bleazard T. Greenstein A. Baimbridge P. Poole D. (2019). Guidelines for conducting research studies with the autism community. Autism Policy & Practice, 2(1 A new beginning), 29–45. PMID: 32226635; PMCID: PMC7099931. 32226635 PMC7099931

[bibr21-23969415251332486] Gray C. A. Garand J. D. (1993). Social stories: Improving responses of students with autism with accurate social information. Focus on Autistic Behavior, 8(1), 1–10. 10.1177/108835769300800101

[bibr22-23969415251332486] Haas K. Costley D. Falkmer M. Richdale A. Sofronoff K. Falkmer T. (2016). Factors influencing the research participation of adults with autism spectrum disorders. Journal of Autism and Developmental Disorders, 46(5), 1793–1805. 10.1007/s10803-016-2708-6 26810436

[bibr23-23969415251332486] Huijnen C. A. G. J. Lexis M. A. S. De Witte L. P. (2017). Robots as new tools in therapy and education for children with autism. International Journal of Neurorehabilitation, 4(4), 278. 10.4172/2376-0281.1000278

[bibr24-23969415251332486] Huijnen C. A. Lexis M. A. Jansens R. De Witte L. P. (2019). Roles, strengths and challenges of using robots in interventions for children with autism spectrum disorder (ASD). Journal of Autism and Developmental Disorders, 49, 11–21. 10.1007/s10803-018-3683-x 30019273

[bibr25-23969415251332486] Huijnen C. A. G. J. Verreussel-Willen H. A. M. D. Lexis M. A. S. de Witte L. P. (2021). Robot KASPAR as mediator in making contact with children with autism: A pilot study. International Journal of Social Robotics, 13(2), 237–249. 10.1007/s12369-020-00633-0

[bibr26-23969415251332486] Kajopoulos J. Wong A. H. Y. Yuen A. W. C. Dung T. A. Kee T. Y. Wykowska A. (2015). Robot-assisted training of joint attention skills in children diagnosed with autism. In Tapus A. André E. Martin J.-C. Ferland F. Ammi M. (Eds.), Social robotics (pp. 296–305). Springer International Publishing. 10.1007/978-3-319-25554-5_30

[bibr27-23969415251332486] Kirby A. V. Bilder D. A. Wiggins L. D. Hughes M. M. Davis J. Hall-Lande J. A. Lee L.-C. McMahon W. M. Bakian A. V. (2022). Sensory features in autism: Findings from a large population-based surveillance system. Autism Research, 15(4), 751–760. 10.1002/aur.2670 35040592 PMC9067163

[bibr28-23969415251332486] Kumazaki H. Muramatsu T. Yoshikawa Y. Matsumoto Y. Kuwata M. Takata K. Ishiguro H. Mimura M. (2022). Differences in the optimal motion of android robots for the ease of communications among individuals with autism spectrum disorders. Frontiers in Psychiatry, 13, 883371. 10.3389/fpsyt.2022.883371 PMC9203835 35722543

[bibr29-23969415251332486] Leekam S. R. Prior M. R. Uljarevic M. (2011). Restricted and repetitive behaviors in autism spectrum disorders: A review of research in the last decade. Psychological Bulletin, 137(4), 562–593. 10.1037/a0023341 21574682

[bibr30-23969415251332486] Lewis K. Hamilton L. G. Vincent J. (2024). Exploring the experiences of autistic pupils through creative research methods: Reflections on a participatory approach. Infant and Child Development, 33(3), e2467. 10.1002/icd.2467

[bibr31-23969415251332486] Louis-Delsoin C. Morales E. Ruiz Rodrigo A. Rousseau J. (2024). Exploring the home environment of adults living with autism spectrum disorder: A qualitative study. International Journal of Developmental Disabilities, 70(2), 213–224. 10.1080/20473869.2022.2071103 38481453 PMC10930097

[bibr32-23969415251332486] MacNeil B. M. Lopes V. A. Minnes P. M. (2009). Anxiety in children and adolescents with autism spectrum disorders. Research in Autism Spectrum Disorders, 3(1), 1–21. 10.1016/j.rasd.2008.06.001

[bibr33-23969415251332486] McKinney A. Weisblatt E. J. Hotson K. L. Ahmed Z. B. Dias C. BenShalom D. Foster J. Murphy S. Villar S. S. Belmonte M. K. (2021). Overcoming hurdles to intervention studies with autistic children with profound communication difficulties and their families. Autism, 25(6), 1627. 10.1177/1362361321998916 33827289 PMC8323331

[bibr34-23969415251332486] Nicolaidis C. Raymaker D. Kapp S. K. Baggs A. Ashkenazy E. McDonald K. Weiner M. Maslak J. Hunter M. Joyce A. (2019). The AASPIRE practice-based guidelines for the inclusion of autistic adults in research as co-researchers and study participants. Autism, 23(8), 2007–2019. 10.1177/1362361319830523 30939892 PMC6776684

[bibr35-23969415251332486] Papagiannopoulou E. A. Chitty K. M. Hermens D. F. Hickie I. B. Lagopoulos J. (2014). A systematic review and meta-analysis of eye-tracking studies in children with autism spectrum disorders. Social Neuroscience, 9(6), 610–632. 10.1080/17470919.2014.934966 24988218

[bibr36-23969415251332486] Pellicano E. Crane L. Gaudion K. (2017). Participatory autism research: A starter pack. UCL Institute of Education. https://issuu.com/crae.ioe/docs/crane-starterpack_pages_v5 .

[bibr37-23969415251332486] Petric F. Miklić D. Cepanec M. Cvitanović P. Kovačić Z. (2017). Functional Imitation Task in the Context of Robot-Assisted Autism Spectrum Disorder Diagnostics: Preliminary Investigations. *2017 26th IEEE International Symposium on Robot and Human Interactive Communication (RO-MAN)*, 1471–1478. 10.1109/ROMAN.2017.8172498

[bibr38-23969415251332486] Petrina N. Carter M. Stephenson J. (2014). The nature of friendship in children with autism spectrum disorders: A systematic review. Research in Autism Spectrum Disorders, 8(2), 111–126. 10.1016/j.rasd.2013.10.016

[bibr39-23969415251332486] Pot E. Monceaux J. Gelin R. Maisonnier B. (2009). Choregraphe: A Graphical Tool for Humanoid Robot Programming. *RO-MAN 2009 - The 18th IEEE International Symposium on Robot and Human Interactive Communication*, 46–51. 10.1109/ROMAN.2009.5326209

[bibr40-23969415251332486] Puglisi A. Caprì T. Pignolo L. Gismondo S. Chilà P. Minutoli R. Marino F. Failla C. Arnao A. A. Tartarisco G. Cerasa A. Pioggia G. (2022). Social humanoid robots for children with autism spectrum disorders: A review of modalities, indications, and pitfalls. Children, 9(7), 953. 10.3390/children9070953 PMC9316169 35883937

[bibr41-23969415251332486] Qi C. H. Barton E. E. Collier M. Lin Y.-L. Montoya C. (2018). A systematic review of effects of social stories interventions for individuals with autism spectrum disorder. Focus on Autism and Other Developmental Disabilities, 33(1), 25–34. 10.1177/1088357615613516

[bibr42-23969415251332486] Rafiee F. Rezvani Habibabadi R. Motaghi M. Yousem D. M. Yousem I. J. (2022). Brain MRI in autism spectrum disorder: Narrative review and recent advances. Journal of Magnetic Resonance Imaging, 55(6), 1613–1624. 10.1002/jmri.27949 34626442

[bibr43-23969415251332486] Rodriguez-Seijas C. Gadow K. D. Rosen T. E. Kim H. Lerner M. D. Eaton N. R. (2020). A transdiagnostic model of psychiatric symptom co-occurrence and autism spectrum disorder. Autism Research, 13(4), 579–590. 10.1002/aur.2228 31647197

[bibr44-23969415251332486] Rosen T. E. Mazefsky C. A. Vasa R. A. Lerner M. D. (2018). Co-occurring psychiatric conditions in autism spectrum disorder. International Review of Psychiatry, 30(1), 40–61. 10.1080/09540261.2018.1450229 29683351

[bibr45-23969415251332486] Rutter M. Bailey A. Lord C. (2003). The social communication questionnaire: Manual. Western Psychological Services.

[bibr46-23969415251332486] Saleh M. A. Hanapiah F. A. Hashim H. (2021). Robot applications for autism: a comprehensive review. Disability and Rehabilitation: Assistive Technology, 16(6), 580–602. 10.1080/17483107.2019.1685016 32706602

[bibr47-23969415251332486] Scassellati B. Admoni H. Matarić M. (2012). Robots for use in autism research. Annual Review of Biomedical Engineering, 14(1), 275–294. 10.1146/annurev-bioeng-071811-150036 22577778

[bibr48-23969415251332486] Short E. S. Deng E. C. Feil-Seifer D. J. Mataric M. J. (2017). Understanding Agency in Interactions Between Children With Autism and Socially Assistive Robots. 10.5898/JHRI.6.3.Short

[bibr49-23969415251332486] Silvera-Tawil D. Roberts-Yates C. (2018). Socially-assistive robots to enhance learning for secondary students with intellectual disabilities and autism. In 2018 27th IEEE International Symposium on Robot and Human Interactive Communication (RO-MAN) (pp. 838–843). IEEE.

[bibr50-23969415251332486] Tziraki M. Garg S. Harrison E. Wright N. B. Hawkes R. Akhtar K. Green J. Stivaros S. (2021). A neuroimaging preparation protocol tailored for autism. Autism Research, 14(1), 65–74. 10.1002/aur.2427 33150732

[bibr51-23969415251332486] van Steensel F. J. A. Bögels S. M. Perrin S. (2011). Anxiety disorders in children and adolescents with autistic spectrum disorders: A meta-analysis. Clinical Child and Family Psychology Review, 14(3), 302–317. 10.1007/s10567-011-0097-0 21735077 PMC3162631

[bibr52-23969415251332486] Wallbridge C. D. McGregor C. Drozdz N. von dem Hagen E. Jones C. R. G. (2023). A systematic review of familiarisation methods used in human–robot interactions for autistic participants. International Journal of Social Robotics, 16(1), 37–53. 10.1007/s12369-023-01015-y

[bibr53-23969415251332486] Wigham S. Rodgers J. South M. McConachie H. Freeston M. (2015). The interplay between sensory processing abnormalities, intolerance of uncertainty, anxiety and restricted and repetitive behaviours in autism spectrum disorder. Journal of Autism and Developmental Disorders, 45(4), 943–952. 10.1007/s10803-014-2248-x 25261248

[bibr54-23969415251332486] Yuan H.-L. Lai C. Y. Y. Wong M. N. K. Kwong T. C. Choy Y. S. Mung S. W. Y. Chan C. C. H. (2022). Interventions for sensory over-responsivity in individuals with autism spectrum disorder: A narrative review. Children (Basel, Switzerland), 9(10), 1584. 10.3390/children9101584 36291519 PMC9601143

