# Supplementary material for: Supporting Autistic Children's Participation in Research Studies: A Mixed-Methods Study of Familiarizing Autistic Children with A Humanoid Robot
Source: Autism Dev Lang Impair. 2025 Apr 24;10:23969415251332486. doi: 10.1177/23969415251332486 (PMC12034964; doi:10.1177/23969415251332486)
Supplement: sj-docx-1-dli-10.1177_23969415251332486 - Supplemental material for Supporting Autistic Children's Participation in Research Studies: A Mixed-Methods Study of Familiarizing Autistic Children with A Humanoid Robot [file sj-docx-1-dli-10.1177_23969415251332486.docx]

# **Supplementary Materials**

Further explanations of the procedures for the familiarisation approaches can be found below. These include the actions performed by both the robot and the researcher.

## **Capability Demonstration**

### ***Wake-Up Sequence***

The first portion of the Capability Demonstration presented the robot’s basic movement, auditory, and light functions in the context of the robot “waking up”. It lasted approximately 70 s.

To mimic the robot being turned off or “asleep”, the robot is seated in a kneeling position on the floor. Its LED lights are turned off, and it does not move or make any noise. The researcher and the child enter the room, and the researcher says, “Thank you for coming in to help us today. This is our robot, Russell. We’ll have you play some games with Russell, then I’ll ask you a few questions at the end when we’re done to see what you thought about him. Does that sound okay?” After the child agrees, the researcher asks, “Is it alright if I turn Russell on now?”

Once the researcher has the child’s consent to begin, the researcher taps the sensor on the robot’s head to trigger the sequence shown in Table S1.

**Table S1**

*Steps of the Wake-Up Sequence*

| Step | Details |
| --- | --- |
| Eye blinking | The robot’s LED eye lights “blink” as if warming up, then turn on permanently. This takes approximately 12 seconds. |
| Musical sequence 1 | The robot plays a short musical sequence (E Major arpeggio – E, G#, B, E), starting quietly and gradually becoming louder. This takes approximately 9 seconds. |
| Head turn | The robot slowly turns its head to the left, then to the right, then looks up, then looks down. This takes approximately 12 seconds. |
| Hand opening | The robot opens and closes both of its hands twice. This takes approximately 4 seconds. |
| Musical sequence 2 with hand movement | The robot plays a second short musical sequence (based on the Westminster Quarters). Whilst the music is playing, the robot slowly raises its arms to its face and rotates its wrists as if rubbing its eyes to wake up. The robot then returns its arms to their initial position and the music stops. This takes approximately 14 seconds. |
| Introduction | The robot raises its right arm, waves, and says, “Hello. My name is Russell. It’s nice to meet you.” It then lowers its arm again. This takes approximately 7 seconds. |
| Consent to proceed | The researcher asks the child if Russell (the robot) can stand up and waits for the child to agree before tapping the robot’s head sensor to trigger the next movement. |
| Stand up | The researcher presses the sensor on the top of the NAO’s head. The robot then says, “I’m going to stand up now – okay?” and stands up. This takes approximately 6 seconds. |

### ***Song and Dance***

The second portion of the Capability Demonstration showed the robot’s full-body movements and more advanced musical and speech capabilities. The robot then performed either “The Wheels on the Bus” (32 s), “The Itsy Bitsy Spider” (30 s), or a stereotypical Robot Dance accompanied by upbeat electronic music (37 s).

The researcher says, “Russell has a little dance he’s been working on, and he’s really excited to show you. You might know the song, so you can dance along if you want to. Can he show you what he’s been practising?” After getting the child’s permission to continue, the researcher presses the sensor on the robot’s head to trigger the Song and Dance.

## **Stimulus and Response**

### ***Option 1: Question and Answer Session***

In this option, the robot asked the child a series of questions. For each question, after the child answered, the robot provided its own answer before asking the next question.

The researcher says, “Now Russell has a few questions he’d like to ask you to get to know you better. He’s really excited to find out about your favourite things. Is that okay?” When the child indicates they are ready to start, the researcher touches the sensor on the top of the robot’s head to begin the activity. The robot asks the following questions, pauses for the child to answer after each one, and does not ask the next question until the researcher on the other side of the one-way mirror prompts it to:

**Robot:** What’s your favourite colour?

**Child:** *[Responds.]*

**Robot:** Cool! I like the colour blue. What is your favourite subject in school?

**Child:** *[Responds.]*

**Robot:** Interesting! I like reading. Do you have any brothers or sisters?

**Child:** *[Responds.]*

**Robot:** Neat! I have one brother and one sister. What is your favourite food?

**Child:** *[Responds.]*

**Robot:** Cool, I like to eat pancakes. How old are you?

**Child:** *[Responds.]*

**Robot:** Wow! I was built four years ago. What is your favourite animal?

**Child:** *[Responds.]*

**Robot:** Cool! My favourite animal is an elephant. What do you want to be when you grow up?

**Child:** *[Responds.]*

**Robot:** Awesome! I want to be a scientist, like my friends. What’s your favourite game to play?

**Child:** *[Responds.]*

**Robot**: Fun! I like playing checkers. Thank you for answering all of my questions.

The researcher was available to clarify the robot’s questions, if required. Parents who selected this option during the Phase 1 interview could choose to have the robot skip over any questions they did not think would be appropriate for their child.

### ***Option 2: Following Game***

In this option, the robot asked the child to complete a series of simple tasks and praised the child after they successfully completed its request.

The researcher says, “Now we have a game the two of you can play. Russell will ask you to do some things for him, and all you have to do is follow along. I’ll play along with you, too. Does that sound okay?” After the child indicates they are ready to begin, the researcher taps the sensor on the top of the robot’s head to start the activity. The robot makes the following requests, and the researcher on the other side of the one-way mirror waits until the child successfully completes the request before triggering the robot to make the next one:

1. Can you clap your hands?
2. Good job! Can you stand up?
3. Awesome! Can you sit back down?
4. Fantastic! Can you pat your head?
5. Great! Can you touch your nose?
6. Hooray! Can you wave at me?

The robot then says, “Great job! Thanks for playing with me!”

The researcher also completed the simple tasks with the child, in case the child was unsure of the instruction. Parents who selected this option during the Phase 1 interview could choose to have the robot skip over any requests they did not think would be appropriate for their child.

### ***Option 3: Mindful Breathing***

This option had the robot lead the child through a mindful breathing exercise. The mindful breathing exercise was developed based on exercises that control the duration of the inhale and exhale, which have been shown to effectively regulate stress responses (Brown et al., 2013).The robot first said that they will take some deep breaths with the child by breathing in for four beats and then out for seven beats. Before the robot and child commenced the breathing exercise together, the robot demonstrated the technique by slowly lifting his arms in front of his chest and counting aloud to four while raising his head, then lowering his arms and counting aloud to seven while tilting his head back down. The robot said that the child could do the arm motions with him, if they wished. The robot and the child repeated this together three times at a speed of 75 beats per minute, lasting about 30 s. Then, the robot led the child in gentle stretches in which they slowly looked up and down, and then left and right. This lasted about 45 s. The researcher completed the activity simultaneously.

## **Static Exploration**

The researcher first explained that the robot needed to rest and is going to take a nap. After getting the child’s permission to continue, the researcher tapped the robot on the head. The robot then said that he was going to sleep, says goodnight, returns to his initial seated position, and his LED lights turn off. This turned the robot’s power off, which meant it would not respond to touch or any other stimuli.

### ***Option 1: Free Exploration***

The researcher invited the child to touch the robot by saying, “Would you like to come a bit closer and touch him? He’s turned off now, so he won’t mind. You’ll just have to be gentle with him.” The duration of this segment was determined by the child.

### ***Option 2: Guided Exploration***

The researcher asked the child to help them give the robot a “check-up”. The researcher explained that they would ask the child to touch a certain body part on the robot; they then demonstrated this process by gently touching the robot’s knee. After confirming that the child understood, the researcher asked the child to touch the robot’s hands, mouth, shoulders, forehead, and elbows in turn. The following script was used:

*While he’s asleep, can you help me give Russell a quick check-up? I think you’ll be really good at it. You can help me by touching different parts of his body when I say their name. So if I ask you to touch his knees, you’ll just touch them like this. [The researcher demonstrates.] Does that sound okay? It would really help me out.*

*Great. Can you touch his hands for me?*

*Good job. Now can you touch his mouth?*

*Wonderful. Can you touch his shoulders?*

*Great. Can you touch his forehead for me?*

*Brilliant. And last, can you touch his elbows for me?*

*Great job, thank you! Everything’s working perfectly. You did great!*

The researcher provided clarification if the child was ever unsure of where the body part was located on the robot.

**References**

Brown, R. P., Gerbarg, P. L., & Muench, F. (2013). Breathing practices for treatment of psychiatric and stress-related medical conditions. *The Psychiatric Clinics of North America*, *36*(1), 121–140. https://doi.org/10.1016/j.psc.2013.01.001
